# Supplementary material for: PixR, a Novel Activator of Conjugative Transfer of IncX4 Resistance Plasmids, Mitigates the Fitness Cost of mcr-1 Carriage in Escherichia coli
Source: mBio. 2022 Jan 4;13(1):e03209-21. doi: 10.1128/mbio.03209-21 (PMC8725589; doi:10.1128/mbio.03209-21)
Supplement: FIG S1 [file mbio.03209-21-sf001.docx]

**Figure S1.** A. Geographic distribution of IncX4 plasmids. Countries where IncX4 plasmids were more prevalent are colored with a darker shade of blue. B. Growth curves of *E. coli* BW25113 carrying pHNSHP23, pHNSHP23∆*mcr-1*, pHNSHP23∆*pixR* and pHNSHP23∆*pixR*∆*mcr-1* mutants. C. In vitro stability of pHNSHP23 and pHNSHP23∆*pixR* in *E. coli* BW25113. D. Protein sequence alignment of PixR and PixR-like proteins. The amino acid sequences of PixR and PixR-like proteins encoded by the representative plasmids were aligned using MUSCLE 3.8.31. PixR1 and PixR2 correspond to homologues encoded by 29 IncX4 plasmids.
