# Supplementary material for: PixR, a Novel Activator of Conjugative Transfer of IncX4 Resistance Plasmids, Mitigates the Fitness Cost of mcr-1 Carriage in Escherichia coli
Source: mBio. 2022 Jan 4;13(1):e03209-21. doi: 10.1128/mbio.03209-21 (PMC8725589; doi:10.1128/mbio.03209-21)
Supplement: FIG S2 [file mbio.03209-21-sf002.docx]

**Figure S2**. Comparative analysis of 178 *mcr-1*-positive IncX4 plasmids from diverse origins found in the Genbank database. The IncX4 plasmid sequences were clustered into 6 groups based on a 65% similarity threshold. The sequences of representative plasmids in each cluster were compared to pHNSHP23 by BRIG.
