# Supplementary material for: PixR, a Novel Activator of Conjugative Transfer of IncX4 Resistance Plasmids, Mitigates the Fitness Cost of mcr-1 Carriage in Escherichia coli
Source: mBio. 2022 Jan 4;13(1):e03209-21. doi: 10.1128/mbio.03209-21 (PMC8725589; doi:10.1128/mbio.03209-21)
Supplement: TABLE S4 [file mbio.03209-21-st004.docx]

**Table S4.** Up- and down-regulated BW25113 KEGG pathways

| Conditions | Up-regulated pathways | Down-regulated pathways | Adj. *p*-value |
| --- | --- | --- | --- |
| Overexpression of *pixR* (pBAD-pixR) *vs* control (pBAD) | **eco03010** Ribosome biosynthesis | none | 2.9E-7 |
|  | **eco00970** Aminoacyl-tRNA biosynthesis | none | 5.7E-4 |
|  | none | **eco00360** Aminoacyl-tRNA biosynthesis | 2.1E-2 |
|  | none | **eco02040** Aminoacyl-tRNA biosynthesis | 9.8E-2 |
