# Supplementary material for: PixR, a Novel Activator of Conjugative Transfer of IncX4 Resistance Plasmids, Mitigates the Fitness Cost of mcr-1 Carriage in Escherichia coli
Source: mBio. 2022 Jan 4;13(1):e03209-21. doi: 10.1128/mbio.03209-21 (PMC8725589; doi:10.1128/mbio.03209-21)
Supplement: TABLE S6 [file mbio.03209-21-st006.docx]

**Table S6.** Comparison of pHNSHP23 conjugative transfer proteins with their respective homologues encoded by other IncX representative plasmids.

| T4SS protein | Amino acid identity（%） | | | | | | | |
| --- | --- | --- | --- | --- | --- | --- | --- | --- |
|  | R485(X1) | R6K(X2) | pEC14_35(X3) | pTi^a^ | pBK31567(X5) | pK55602_2(X6) | P3(X7) | pCAV1043-58(X8) |
| TaxA | 24.75% | 24.52% | 25.24% | NA | 29.73% | 25.44% | 81.55% | 29.73% |
| TaxC | 35.39% | 33.69% | 35.03% | NA | 35.59% | 35% | 84.15% | 35.59% |
| Eex | 50.77% | NA | 33% | NA | 45.59% | 43.28% | NA | 48.57% |
| PilX1/VirB1 | 54.69% | 52% | 51% | 32% | 53% | 58.85% | 68.42% | 53% |
| PilX2/VirB2 | 30.86% | 32.53% | 33.33% | NA | 36.14% | 37.84% | NA | 36.14% |
| PilX3-4/VirB3-4 | 42.11% | 41.77% | 42.42% | 30% | 41.98% | 49.95% | 82.97% | 41.77% |
| PilX5/VirB5 | 42.86% | 33.18% | 41% | NA | 32.63% | 31.93% | 74.58% | 32.63% |
| PilX6/VirB6 | 36.54% | 38.74% | 36.07% | NA | 38.02% | 38.8% | 81.1% | 37.72% |
| PilX7/VirB7 | 35.90% | 33.33% | NA | 34% | 33.33% | 38.46% | 65.12 | 33.33% |
| PilX8/VirB8 | 42.68% | 37.93% | 40.91% | 29% | 44.13% | 41.74% | 82.97% | 41.92% |
| PilX9/VirB9 | 40.72% | 40.92% | 40.85% | 25% | 41.69% | 45.95% | 73.09% | 40.52% |
| PilX10/VirB10 | 40.80% | 38.78% | 42.77% | 38% | 37.05% | 43.7% | 70.41% | 37.71% |
| PilX11/VirB11 | 43.6% | 41.77% | 40.81% | 34% | 41.74% | 52.13% | 82.51% | 42.06% |
| TaxB | 40.17% | 38.23% | 39.35% | NA | 39.15% | 41.47% | 87.44% | 39.32% |

^a^pTi carried the *virB* operon

NA, not applicable
