# Supplementary material for: PixR, a Novel Activator of Conjugative Transfer of IncX4 Resistance Plasmids, Mitigates the Fitness Cost of mcr-1 Carriage in Escherichia coli
Source: mBio. 2022 Jan 4;13(1):e03209-21. doi: 10.1128/mbio.03209-21 (PMC8725589; doi:10.1128/mbio.03209-21)
Supplement: TEXT S1 [file mbio.03209-21-s0001.docx]

**Supplementary Material**

**PixR, a novel activator of conjugative transfer of IncX4 resistance plasmids, mitigates the fitness cost of *mcr-1* carriage** **in *Escherichia coli***

Lingxian Yi^1,2#^, Romain Durand^3a#^, Frédéric Grenier^3^, Jun Yang^1,2^, Kaiyang Yu^1,2^, Vincent Burrus^3^* and Jian-Hua Liu^1,2^*

^1^College of Veterinary Medicine, Key Laboratory of Zoonosis of Ministry of Agricultural and Rural Affairs, Guangdong Provincial Key Laboratory of Veterinary Pharmaceutics Development and Safety Evaluation, South China Agricultural University, Guangzhou 510642, China

^2^Guangdong Laboratory for Lingnan Modern Agriculture, Guangzhou, China

^3^Département de biologie, Université de Sherbrooke, Sherbrooke J1K 2R1, Québec, Canada

^#^These authors contributed equally to this work

* To whom correspondence should be addressed

Email: [vincent.burrus@usherbrooke.ca](mailto:vincent.burrus@usherbrooke.ca); [jhliu@scau.edu.cn](mailto:jhliu@scau.edu.cn)

^a^Current Address: Institut de Biologie Intégrative et des Systèmes, Université Laval, Québec, Canada

**Text S1. MATERIALS AND METHODS**

**Bacterial strains, plasmids and culture conditions**

*E. coli* was cultured under static conditions on LB agar medium or with shaking (220 rpm) in LB broth, with the appropriate antibiotics. In complementation assays, to induce expression from pBAD30 plasmid, cells were cultivated in LB broth supplemented with 0.2% L-arabinose and the appropriate antibiotics. Antibiotics were used at the following concentrations: colistin (Cl), 2 μg·mL^-1^; ampicillin (Ap), 100 μg·mL^-1^; kanamycin (Km), 50 μg·mL^-1^; chloramphenicol (Cm), 30 μg·mL^-1^; streptomycin (Sm), 3 000 μg·mL^-1^.

**Plasmids and strains constructions**

Deletion mutants were constructed in *E. coli* BW25113 via homologous recombination following the previously described method (1). Briefly, the chloramphenicol resistance gene *cat* flanked by FLP recombination target (FRT) sites was amplified from plasmid pKD3 with primers *pixR*_F/R, cds4_F/R, cds9_F/R, cds16_F/R or mcr-d-F/R. The PCR products were purified and electroporated into *E. coli* BW25113 bearing pKD46 and pHNSHP23 to yield the deletion mutants pHNSHP23Δ*pixR::cat*, pHNSHP23Δ*cds4::cat*, pHNSHP23Δ*cds9::cat*, pHNSHP23Δ*cds16::cat*, and BW25113/pHNSHP23Δ*mcr-1::cat*. The mutant colonies were selected on LB agar with 30 µg/mL chloramphenicol at 37℃ to lose the thermosensitive plasmid pKD46. The chloramphenicol resistance marker was removed from deleted mutant BW25113/pHNSHP23Δ*pixR::cat*, pHNSHP23Δ*cds4::cat*, pHNSHP23Δ*cds9::cat*, pHNSHP23Δ*cds16**::cat*, and BW25113/pHNSHP23Δ*mcr-::cat* using pCP20 to obtain mutant BW25113/pHNSHP23Δ*pixR*, pHNSHP23Δ*cds4*, pHNSHP23Δ*cds9*, pHNSHP23Δ*cds16* and BW25113/pHNSHP23Δ*mcr-1* (1). The resulting deletion mutants were further confirmed by sequencing with primer pairs *pixR*C_F/R, cds4-c-F/R, cds9-c-F/R, cds16-c-F/R or mcr-c-F/R. The mutant pHNSHP23Δ*pixR*Δ*mcr-1::cat* was constructed the same way by using BW25113 bearing pHNSHP23Δ*pixR.* The wild-type plasmid pHNSHP23 and its deletion mutant Δ*pixR* were electroporated into *E. coli* GED8P261. The open reading frame (ORF) of *pixR* and *cds9* was amplified with primers BAD-*pixR*_F/R and BAD-cds9_F/R. The PCR product was purified, digested with EcoRI and SalI, and ligated into the expression plasmid pBAD30 using the ClonExpress II one-step cloning kit (Vazyme Biotech, Piscataway, NJ, USA) to obtain plasmid pBAD-*pixR* and pBAD-*cds9*. Primer pair BAD-F/R was used to confirm the constructed vector. pBAD-*pixR* and pBAD30 were transformed into *E. coli* BW25113/pHNSHP23Δ*pixR*. *pixR* and *cds9* with their native promoters were amplified and cloned into pHSG575 in the similar way by using primer HSGpixR-F/R and HSGcds9-F/R, and the complementation vectors were confirmed with primer HSG575-F/R. A 120 bp fragment was amplified from pHNSHP23 with primers pilXpromoter-F/R and cloned upstream a promoterless *lacZ* gene to produce *P_pilX_-lacZ*. The pET28b recombinant plasmid was constructed with a similar approach using primers 28b-pixR_F/R. *pixR* tagged with a 6His C-terminal epitope was cloned into the expression vector pET28b, then electroporated into *E. coli* BL21.

**Bacterial conjugation experiments**

Streptomycin-resistant *E. coli* C600 was used as the recipient strain. *E. coli* BW25113 and swine *E. coli* GED8P261 containing pHNSHP23 or its derivatives were used as donor strains. For broth mating, after the OD_600_ of the donor and recipient cultures reached 0.8, equal volumes (1 mL) of each culture were mixed, and the suspension was incubated at 37 ºC overnight without shaking. For solid mating, 1mL of the mixed cultures was centrifuged at 3 000 g for 5 minutes, and supernatants were discarded. Cell pellets were washed with 1 volume of LB medium, then resuspended in 100 µl LB and dropped on LB agar plates, which were incubated at 37 ºC for 3 h. Transconjugants were selected on LB agar plates containing 2 μg·mL^-1^ colistin and 3 000 μg·mL^-1^ streptomycin. Plasmid transfer frequencies were calculated as the ratio of transconjugant over recipient CFUs.

**Comparative analysis**

A BLASTn search (<https://blast.ncbi.nlm.nih.gov/Blast.cgi>) for all complete IncX4 plasmid sequences in the NCBI Genbank database was conducted using the *repA* sequences of IncX4 plasmids extracted from the PlasmidFinder database (<https://bitbucket.org/genomicepidemiology/plasmidfinder_db/src/master/>). A total of 271 IncX4 plasmid sequences were collected (Table S1). BLASTp and Pfam (<http://pfam.xfam.org>) were used to analyze the PixR protein. Protein sequences were aligned using MUSCLE 3.8.31 (2). Gene organization diagrams were generated with Easyfig 2.2.2. The *mcr-1* positive IncX4 plasmid sequences were compared with pHNSHP23 sequence by using CD-HIT (<http://weizhong-lab.ucsd.edu/cd-hit/>) and BRIG (<http://sourceforge.net/projects/brig>).

**Cappable-seq and RNA-seq assays**

Overnight cultures of BW25113/pHNSHP23, BW25113/pHNSHP23Δ*pixR*, BW25113/pHNSHP23Δ*pixR* + pBAD30 and BW25113/ pHNSHP23Δ*pixR* + pBAD-*pixR* were diluted to an OD_600_ of 0.05 in 50 mL LB supplemented with the appropriate antibiotics. Cultures were grown at 37°C until the OD_600_ reached 0.6, at which point total RNA was extracted. For strains containing pBAD vectors, cultures were grown to an OD_600_ of 0.3, then induced with 0.2% of arabinose for 2 hours until OD_600_ reached 0.6. Total RNA was extracted using the Direct-zol RNA MiniPrep kit (Zymo Research) according to the manufacturer’s instructions, with the recommended DNase I treatment. Cappable-seq was performed as previously described from 10 µg of total RNA (3). For RNA-seq assays, 1 µg of total RNA was fragmented in 5 volumes of RNA Fragmentation Buffer (200 mM Tris-Acetate pH=8.1, 500mM KOAc, 150mM MgOAc) by incubating at 95 ºC for 5 minutes, then quenching on ice immediately. RNA was purified using the RNA Clean & concentrator-5 kit (Zymo Research), according to the manufacturer’s instructions. All samples were evaluated before and after fragmentation using a 2100 Bioanalyzer instrument (Agilent Technologies).

**High-density transposon mutagenesis (HDTM) assays**

The HDTM assays were conducted as previously described (4). Briefly, plasmid pFG051 was transferred by conjugation from *E. coli* MFD*pir*^+^ to BW25113/pHNSHP23 by incubating 2 h on LB agar plates supplemented with 0.3 mM diaminopimelate (DAP) at 30ºC in duplicates. The mating cultures were collected in 25 mL LB broth and then spread on 20 large LB agar plates (150mm) with selective antibiotics. After overnight incubation at 37ºC, the colonies present on the plates were collected and suspended in 25 mL LB, designated as the ‘Input library’. 1 mL of ‘Input library’ culture was refreshed in 25 mL of LB broth supplemented with the appropriate antibiotics to perform two successive rounds of conjugation assays using strains VB112 and VB111 as recipients.

**Illumina sequencing library preparation**

Cappable-seq and RNA-seq libraries were prepared using the NEBNext Small RNA Library Prep Set for Illumina (NEB) with the following modification (5). The NEBNext 5’ SR Adaptor for Illumina was replaced by primer 5’-hybrid-A0 oligo (5). All libraries were amplified and barcoded in a qPCR machine with Veraseq 2.0 High-Fidelity DNA polymerase (Enzymatics).

Illumina sequencing libraries were prepared as previously described (6). An aliquot of mutant library culture was used to extract the genomic DNA with Quick gDNA Miniprep kit (ZymoResearch). The purified DNA was fragmented, end-repaired, A-tailed, and ligated using NEBNext Ultra II FS DNA Library Prep Kit (New England Biolabs) with our Nextera-B adaptator (6). The appropriate size of DNA was selected using DNA Ampure XP beads (Agencourt) with a 0.8 ratio and then were barcoded by qPCR for Illumina sequencing.

The quality and size distribution of all amplified libraries were checked on the Agilent 2100 Bioanalyzer. Illumina sequencing was performed on NextSeq 500/550 High Output system at the Plateforme Rnomique de I’Université de Sherbrooke (Sherbrooke, QC, Canada).

**Bioinformatic analyses**

Reads were trimmed with Trimmomatic to discard nucleotides with a quality score below 30 and reads with a length below 36 bp (7). Quality was assessed before and after using FastQC (8). Trimmed reads were aligned on the *E. coli* BW25113 genome (CP009273) and pHNSHP23 (MF774184) using Bowtie 2 (9). Alignment quality was assessed using SAMStat (10), and reads with a quality score below 10 were discarded using SAMtools view. Reads were then compressed, sorted, and indexed using SAMtools (11). Cappable-seq reads were chopped to their first nucleotide and density was calculated separately for each DNA strand using BEDTools genomecov (12). Density files were ultimately compressed to bigWig format and visualized on the UCSC Genome Browser. RPKM values were calculated for each DNA strand separately, using a script adapted from EDGE-pro (13). Differential expression analysis was performed using DESeq2 (14). Pathway enrichment analysis was performed as described previously using GAGE (3, 15).

HDTM data were analyzed as previously described (6). First, the reads were trimmed using Trimmomatic version 0.36 with the parameters SLIDINGWINDOW:4:20 MINLEN:20 ILLUMINACLIP:2:30:15 (7). The quality of the reads was assessed with FastQC version 0.11.5 before and after trimming. The reads were aligned with BWA MEM version 0.7.15 using default parameters. SAMtools version 1.3.1 was used to generate alignments statistics and to discard low-quality alignments (MAPQ < 10) or multiple alignments (11). We used sam2sites.py with the parameters –read_len_threshold 20 and –score_threshold 0 to get the exact positions of the transposon insertions. The insertions sites files were then encoded in bigWig format using the Kent utilities (16). To get gene level insertion statistics, we used BEDTools intersect version 2.26.0 together with sites2genes.py (12). The python scripts sam2sites.py and sites2genes.py are available at https://github.com/fredericQC/HDTM_tools.

Essential conjugation factors were identified by using the following formula: F index = (insertion index × read counts) Input library/(insertion index ×read counts)output library, with the insertion index being the ratio of insertion counts over gene length. The *pilX,* *taxAC*, and *taxB* genes were used to set the threshold. Genes were considered essential when their F index was superior to 100.

**Reverse transcription and qPCR**

Overnight cultures of C600, BW25113/pHNSHP23, BW25113/pHNSHP23ΔpixR, BW25113/pHNSHP23ΔpixR + pBAD30 and BW25113/pHNSHP23ΔpixR + pBAD-pixR were diluted to an OD600 of 0.05 in 2 mL LB containing the appropriate antibiotics. The cells were grown at 37°C until the OD600 reached 0.8 with 0.2% arabinose to induce PBAD. Equal volumes (1 mL) of donor BW25113/pHNSHP23 and recipient C600 cultures were mixed, and the suspension was incubated at 37 ºC for 30 mins without shaking. Total RNA was extracted by using the Hipure Bacterial RNA Kit (Magen, China). Reverse transcription was carried out with 1 µg RNA using TB Green Premix Ex Taq™ II (Takara, Japan). Targets of around 110 bp were amplified with primers qtaxB-F/R, qpilX11-F/R, qpilX3-4-F/R, and qtrbM -F/R by qPCR, while 16S or BW25113 specific chromosomal genes *yahJ* was used as internal control with primers 16S-F/R or yahJ-F/R. Relative expression was estimated by the 2-ΔΔCt method. The experiment was performed with three biological replicates.

A reverse transcription experiment was performed using a primer located at the 3’ end of trbM and total RNA extracted from BW25113 cells bearing pHNSHP23. A PCR amplification using the reverse cDNA with primers hyp9-pilX1R/F and trbM-pilX11-R/F was performed to confirm that cds9, trbM, and taxB are indeed part of the pilX operon.

**Protein expression and purification**

An overnight culture of *E coli* BL21/pET28b-*pixR*-CHis was refreshed 1:100 (vol/vol) in LB broth with kanamycin until it reached an OD_600_ of 0.1 and then was induced overnight with 1 mM of IPTG at 25°C. Cells were collected and resuspended in lysis buffer (50 mM potassium phosphate buffer, 300 mM NaCl, 5mM imidazole, and a tablet of protease inhibitor cocktail (Sigma-Aldrich, St. Louis, MO, USA), pH 8.0). The cells were lysed by sonication (90 cycles, 10s ON/10s OFF), then centrifuged at ~9,500 g for 15 min. The supernatant was collected and incubated with Ni-NTA resin (Bioteke Corporation, Haimen, Jiangsu, China) according to the manufacturer’s instructions. The protein concentration was measured using the Bi Yuntian BCA assay kit (Bioteke Corporation, Haimen, Jiangsu, China).

**Electrophoretic Mobility Shift Assay (EMSA)**

The EMSA assay was performed using an Electrophoretic Mobility Shift Assay kit (Thermo Fisher Scientific, E33075) according to the manufacturer’s instructions. A ~120 bp fragment encompassing the promoter of the *pilX* operon was amplified using primers EMSA-pixR-F/R and EMSA-puc19-F/R, then incubated with 5 volumes of binding buffer provided with the kit. The final mixtures were run on a 6% non-denaturing PAGE gel for 1h at 200V. The gel was stained with SYBR Green and then imaged with a Bio-Rad Molecular Imager Chemi Doc XRS+ system.

**β-galactosidase assay**

β-Galactosidase assays were performed as previously described (17). Cultures were grown overnight in LB broth supplemented with the appropriate antibiotics and refreshed in LB broth. After the OD_600_ reached 0.2, the cultures were induced by adding 0.2% arabinose and incubating for 2 h at 37 ºC.

**References**

1. Datsenko KA, Wanner BL. 2000. One-step inactivation of chromosomal genes in Escherichia coli K-12 using PCR products. Proc Natl Acad Sci U S A 97:6640-5.

2. Edgar RC. 2004. MUSCLE: a multiple sequence alignment method with reduced time and space complexity. BMC Bioinformatics 5:113.

3. Durand R, Huguet KT, Rivard N, Carraro N, Rodrigue S, Burrus V. 2021. Crucial role of Salmonella genomic island 1 master activator in the parasitism of IncC plasmids. Nucleic Acids Res doi:10.1093/nar/gkab204.

4. Neil K, Allard N, Grenier F, Burrus V, Rodrigue S. 2020. Highly efficient gene transfer in the mouse gut microbiota is enabled by the Incl2 conjugative plasmid TP114. Commun Biol 3:523.

5. Carraro N, Matteau D, Luo P, Rodrigue S, Burrus V. 2014. The master activator of IncA/C conjugative plasmids stimulates genomic islands and multidrug resistance dissemination. PLoS Genet 10:e1004714.

6. Roy D, Huguet KT, Grenier F, Burrus V. 2020. IncC conjugative plasmids and SXT/R391 elements repair double-strand breaks caused by CRISPR-Cas during conjugation. Nucleic Acids Res 48:8815-8827.

7. Bolger AM, Lohse M, Usadel B. 2014. Trimmomatic: a flexible trimmer for Illumina sequence data. Bioinformatics 30:2114-20.

8. Andrews S. 2010. FastQC: A Quality Control tool for High Throughput Sequence Data. <https://www.bioinformatics.babraham.ac.uk/projects/fastqc/>. Accessed

9. Langmead B, Salzberg SL. 2012. Fast gapped-read alignment with Bowtie 2. Nat Methods 9:357-9.

10. Lassmann T, Hayashizaki Y, Daub CO. 2011. SAMStat: monitoring biases in next generation sequencing data. Bioinformatics 27:130-1.

11. Li H, Handsaker B, Wysoker A, Fennell T, Ruan J, Homer N, Marth G, Abecasis G, Durbin R, Genome Project Data Processing S. 2009. The Sequence Alignment/Map format and SAMtools. Bioinformatics 25:2078-9.

12. Quinlan AR, Hall IM. 2010. BEDTools: a flexible suite of utilities for comparing genomic features. Bioinformatics 26:841-2.

13. Magoc T, Wood D, Salzberg SL. 2013. EDGE-pro: Estimated Degree of Gene Expression in Prokaryotic Genomes. Evol Bioinform Online 9:127-36.

14. Love MI, Huber W, Anders S. 2014. Moderated estimation of fold change and dispersion for RNA-seq data with DESeq2. Genome Biol 15:550.

15. Luo W, Friedman MS, Shedden K, Hankenson KD, Woolf PJ. 2009. GAGE: generally applicable gene set enrichment for pathway analysis. BMC Bioinformatics 10:161.

16. Rhead B, Karolchik D, Kuhn RM, Hinrichs AS, Zweig AS, Fujita PA, Diekhans M, Smith KE, Rosenbloom KR, Raney BJ, Pohl A, Pheasant M, Meyer LR, Learned K, Hsu F, Hillman-Jackson J, Harte RA, Giardine B, Dreszer TR, Clawson H, Barber GP, Haussler D, Kent WJ. 2010. The UCSC Genome Browser database: update 2010. Nucleic Acids Res 38:D613-9.

17. Malke H. 1993. Jeffrey H. Miller, A Short Course in Bacterial Genetics – A Laboratory Manual and Handbook for Escherichia coli and Related Bacteria. Cold Spring Harbor 1992. Cold Spring Harbor Laboratory Press. ISBN: 0–87969-349–5. Journal of Basic Microbiology 33:278-278.
