# Supplementary material for: PixR, a Novel Activator of Conjugative Transfer of IncX4 Resistance Plasmids, Mitigates the Fitness Cost of mcr-1 Carriage in Escherichia coli
Source: mBio. 2022 Jan 4;13(1):e03209-21. doi: 10.1128/mbio.03209-21 (PMC8725589; doi:10.1128/mbio.03209-21)
Supplement: TABLE S2 [file mbio.03209-21-st002.docx]

**Table S2.** Strains and plasmids used in this study

| Strain or Plasmid | Relevant genotype or phenotype | Reference |
| --- | --- | --- |
| *Escherichia coli* |  |  |
| BW25113 | F^_^Δ(araD-araB)567 ΔlacZ4787(::rrnB-3) λ^_^rph-1 Δ(rhaD-rhaB)568 hsdR514 | (1) |
| VB111 | Nx derivative of MG1655(Nx^R^) | (2) |
| VB112 | Rf derivative of MG1655 (Rf^R^) | (2) |
| BL21 | F-*ompT* *hsdSB*(rB- mB-) gal dcm λ(DE3) Ω PtacUV5::T7 polymerase | Novagen |
| C600 | (Sm^R^) | (3) |
| MFDpir+ | MG1655 RP4-2-Tc::[ΔMu1::*aac(3)IV*-Δ*aphA*-Δ*nic35*-ΔMu2::*zeo*] Δ*dapA*::(*erm-pir*) ΔrecA | (4) |
| GDE8P261 | *E. coli* isolated from swine at slaughter in Guangzhou, China | (5) |
| Plasmid |  |  |
| pBAD30 | ori_p15A_*bla* *araC* P_BAD_ (Ap^R^) | (6) |
| pKD3 | Cm^R^ PCR template for one-step chromosomal gene inactivation | (1) |
| pKD4 | Km^R^, PCR template for one-step chromosomal gene inactivation | (1) |
| pCP20 | Thermo-inducible expression of Flp recombinase (Ts^R^ Ap^R^ Cm^R^) | (7) |
| pKD46 | Ap^R^, λRed recombinase expression | (1) |
| pBAD-*pixR* | pBAD30::*pixR* (Ap^R^) | This study |
| pBAD-*cds9* | pBAD30::*cds9* (Ap^R^) | This study |
| pET28b | Km^R^, lacI^q^ | Novagen |
| pET28b-pilxR | pET28b::*pixR* (Km^R^) | This study |
| pOPlacZ | pAH56 lacZ (Km^R^) | (8) |
| pFG036 | *ori*_pMB1_, *cI857* (Ts^R^) repressor, *tetM* (Tc^R^) | Addgene #137996 |
| pFG051 | *ori*_R6K_, Tn5 *tnp* under λPL promoter, *oriT*_RP4_, Tn5*d-aadA7* (Sp^R^) | Addgene #137997 |
| pHSG575 | *oriV*_pSC101_*(*Cm^R^*)* | (9) |
| pHSG575-*cds9* | pHSG575 carrying *cds9* gene with native promoter | This study |
| pHSG575-*pixR* | pHSG474 carrying *pixR* with native promoter | This study |

Ap, ampicillin; Cm, chloramphenicol; Km, kanamycin; Nx, nalidixic acid; Rf, rifampicin; Sm, streptomycin; Sp, spectinomycin; Tc, tetracycline; Ts, thermosensitive.

**References**

1. Datsenko KA, Wanner BL. 2000. One-step inactivation of chromosomal genes in Escherichia coli K-12 using PCR products. Proc Natl Acad Sci U S A 97:6640-5.

2. Ceccarelli D, Daccord A, Rene M, Burrus V. 2008. Identification of the origin of transfer (oriT) and a new gene required for mobilization of the SXT/R391 family of integrating conjugative elements. J Bacteriol 190:5328-38.

3. Wang J, Yao X, Luo J, Lv L, Zeng Z, Liu JH. 2018. Emergence of Escherichia coli co-producing NDM-1 and KPC-2 carbapenemases from a retail vegetable, China. J Antimicrob Chemother 73:252-254.

4. Ferrieres L, Hemery G, Nham T, Guerout AM, Mazel D, Beloin C, Ghigo JM. 2010. Silent mischief: bacteriophage Mu insertions contaminate products of Escherichia coli random mutagenesis performed using suicidal transposon delivery plasmids mobilized by broad-host-range RP4 conjugative machinery. J Bacteriol 192:6418-27.

5. Liu YY, Zhou Q, He W, Lin Q, Yang J, Liu JH. 2020. mcr-1 and plasmid prevalence in Escherichia coli from livestock. Lancet Infect Dis 20:1126.

6. Guzman LM, Belin D, Carson MJ, Beckwith J. 1995. Tight regulation, modulation, and high-level expression by vectors containing the arabinose PBAD promoter. J Bacteriol 177:4121-30.

7. Cherepanov PP, Wackernagel W. 1995. Gene disruption in Escherichia coli: TcR and KmR cassettes with the option of Flp-catalyzed excision of the antibiotic-resistance determinant. Gene 158:9-14.

8. Carraro N, Matteau D, Luo P, Rodrigue S, Burrus V. 2014. The master activator of IncA/C conjugative plasmids stimulates genomic islands and multidrug resistance dissemination. PLoS Genet 10:e1004714.

9. Takeshita S, Sato M, Toba M, Masahashi W, Hashimoto-Gotoh T. 1987. High-copy-number and low-copy-number plasmid vectors for lacZ alpha-complementation and chloramphenicol- or kanamycin-resistance selection. Gene 61:63-74.
