# Supplementary material for: PixR, a Novel Activator of Conjugative Transfer of IncX4 Resistance Plasmids, Mitigates the Fitness Cost of mcr-1 Carriage in Escherichia coli
Source: mBio. 2022 Jan 4;13(1):e03209-21. doi: 10.1128/mbio.03209-21 (PMC8725589; doi:10.1128/mbio.03209-21)
Supplement: TABLE S3 [file mbio.03209-21-st003.docx]

**Table S3.** Oligonucleotides used in this study.

| Primers | Sequence |
| --- | --- |
| pixR_F | CTCACCTGCTGATCACAACAACCTACGAGGGAGGTTATCGTGTAGGCTGGAGCTGCTTC |
| pixR_R | AAGCGTACATCAGAATAATGTCTTTTTGTTGCTCACTCATCCATATGAATATCCTCCTT |
| pixRc_F | TAATTTGATTTTGGCGCAGGT |
| pixRc_R | TTCTGATCCCTGCCAACGAGA |
| mcr-d-f | GAGAAACTACTCAAAAAATAAACGGTGGGATAATTGCGGTGTAGGCTGGAGCTGCTTCG |
| mcr-d-r | ACGCCCAAGCGATCCAGCGTATCCAGCACATTTTCTTGGTCCATATGAATATCCTCCTT |
| mcr-c-F | GCGACCATCAGATCAATCTGACT |
| mcr-c-R | CGCAATTATCCCACCGTTT |
| BAD-pixR_F | GTCAGAATTCAAGGAGGATAATAAATGACTGAGCATGATGCTATCTGCAT |
| BAD-pixR_R | CACTGAGTCGACTCATAAATTCCCCCTCTCACTGAT |
| BAD-cds9_F | GTCAGAATTCAAGGAGGATAATAAATGAAGGAGAAATCAGATAAGTCATC |
| BAD-cds9_R | CACTGAGTCGACCTAACGCTCCATATATCCATCAAT |
| BAD_F | ATTTGCACGGCGTCACACTT |
| BAD_R | GACCGCTTCTGCGTTCTGAT |
| 28b-pixR_F | TTTAAGAAGGAGATATACCATGGGCATGACTGAGCATGATGCTATCTGC |
| 28b-pixR_R | GCTCGAGTGCGGCCGCAAGCTTTCAGTGGTGGTGGTGGTGGTGTAAATTCCCCCTCTCACTGA |
| 28b-pixR_cf | GGCTTTGTTAGCAGCCGGATCTC |
| 28b-pixR_cr | TTGTTTAACTTTAAGAAGGAGAT |
| PpixR_f | AGAAGTAAAAAGTGTGGATTG |
| PpixR_r | TTAATCAATCCACTATAAGTGT |
| Probe for IncX4 | ATGAGAATGACGACAAATAAGACTTCCCTTTCTCGCTTAACAAAAGTGAGACATCGAAATGAATTGAATTCAACGCTGTCAACATTGCCTATGGCTGCTAAAAAAGTCTTATTTTTAGCCATGTGCCAGATAAACTCTAAAAATGAATTCGATGATGATCACATATTTTATGTGACAGTTGCCGATTACATTAAATGGGTTCAGGTTAAGCCTGATGCGGCTTATCTTGCTTTGAGGGATGGCTCTAATATATTAGATACGACGCTTCTTAAGCTGAAACATGATGAAATATTAGAACTGAGTAGTGATTTGGGATTTAAGTTCACTAAAAGTAATGTACCTGATTCAATGAATTTGAGTCTGACTGTTTTTTC |
|  | ATGGTCTTAAAGAATAATAAAAATAGCGACTGTAATGATGTTCAAAGTTTGTTGGCACAGGGAAATCAATTGCTGGAAGGTGCATACGATATAACGCTAATTGAAATGCGTCTTCTGTATCTGGCATTGACTAAGATTGATAGCCGTAAACCGCAGCCTGCAAATGAATATACATTGTTTGCGAAGGAATACAGGGACACTTTTTCTTTAGATTCGAAAAATTGTTATGAACAGTTAAAATCTGCTGCGAGTTCT |
| pilXpromoter-F | CCGGTAGTCAATAAACCGGTGTTAATCAATCCACTATAAGTGTG |
| pilXpromoter-R | AGTGCCAAGCTTGTCGACGGATCAATCATGGTCATACGAATCATAATATGACCAT |
| 16s-F- | TGTAGCGGTGAAATGCGTAGA |
| 16s-R | CACCTGAGCGTCAGTCTTCGT |
| qpixR-q-F | TTGACACGAGAAGGGCATTAGTC |
| qpixR-q-R | GACTAAAGCCTTCAACTGTCAGAAAA |
| qpilx11-F | CGTTGCTCCGGCAAATACA |
| qpilx11-R | CGATGATGAGCTTCTTGATTTATTCA |
| qpilx3-4-F | TAGCCCATTGGGACAAACG |
| qpilx3-4-R | TGGCGAAATGCGGGAAT |
| qtaxB-F | GCACCTTTACCAGCCCTTGTT |
| qtaxB-R | GGTGACGAAAAAGACGGGATT |
| qtrbM-F | ATCCTTTTCAGCACTTCTGCATT |
| qtrbM-R | CTTGCGCTTCGGTACTTTGC |
| IncI2-F | CTGTCGGCATGTCTGTCTC |
| IncI2-R | CTGGCTACCAGTTGCTCTAA |
| trbm-pilx11-F | TTAACCTCTGACACGACCAA |
| trbm-pilx11-R | CGGCTCTATCATAACAGCAG |
| hy9-pilx1-R | ATGAAGGAGAAATCAGATAAG |
| hy9-pilx1-F | CTCTGATAACTGTTAGAGCTTCCTCCTT |
| cds4-F | TATACATTAGTGTGAAACAATCAGATTAGAAGGTACATCATGGGAATTAGCCATGGTCC |
| cds4-R | AAGAAAATGATAAAGAGTTTATTGGGCCACTTGCTATGATGTAGGCTGGAGCTGCTTCG |
| cds16-F | AAGACGGGAACAGCAGCCACGTAGCAAAAAGGGAAACAGATGGGAATTAGCCATGGTCC |
| cds16-R | TTTTCGCTCGATATCGGCTGCAATATCAACGGGATTCCGTGTAGGCTGGAGCTGCTTCG |
| cds9-F | AGATGTTTTCGTTTTCTCTTCGATAAATGCATTCAACCTGTGTAGGCTGGAGCTGCTTC |
| cds9-R | ATGAAGGAGAAATCAGATAAGTCATCGGAAAAAGGGGTTTCCATATGAATATCCTCCTT |
| cds4-c-F | GCTCCTAAATACCCGTGCAA |
| cds4-c-R | TCCGGGCCTTTGTAAGAGCA |
| cds16-C-F | TTTCGTAGTTTTGTTCCCGAT |
| cds16-C-R | GCAGGGCGACAGGGTGGCAA |
| cds9-C-F | GCCGCACACAGCAACAAGGCT |
| cds9-C-R | ACGAATCATAATATGACCAT |
| HSGcds9-F | ACGCGTCGACCTAACGCTCCATATATCCATCAA |
| HSGcds9-R | CCGGAATTCATTCGATTAACTAATGTTTCA |
| HSGpixR-R | CCGGAATTCCAGAAAACATGAGCAAGGTCA |
| HSGpixR-F | ACGCGTCGACTCATAAATTCCCCCTCTCACTGAT |
| HSG575-F | TGCTTCCGGCTCGTATGTTGTGTGGAATTG |
| HSG575-R | CAGGGTTTTCCCAGTCACGACGTTGTAAAA |
| IncX4-F | AGCAAACAGGGAAAGGAGAAGAT |
| IncX4-R | CTGTCGGCATGTCTGTCTC |
| yahJ-F | CGGTAGCTGGCACCCTAAAC |
| yahJ-R | GCAGCACGTTATCGAGATAGTAATG |
